# Investigation of Toughening Mechanisms in Elastomeric Polycarbonate Blends through Morphological and Mechanical Characterization at Small and Medium Strain Rates

**DOI:** 10.3390/polym16162303

**Published:** 2024-08-15

**Authors:** Pedro Veiga Rodrigues, Bruno Ramoa, Maria Cidália R. Castro, Ana Vera Machado

**Affiliations:** Department of Polymer Engineering, Institute for Polymers and Composites (IPC), University of Minho, 4804-533 Guimarães, Portugal; bruno.ramoa@dep.uminho.pt (B.R.); cidaliacastro@dep.uminho.pt (M.C.R.C.); avm@dep.uminho.pt (A.V.M.)

**Keywords:** polycarbonate elastomeric blends, toughening mechanism, small strain rate, medium strain rate

## Abstract

Despite polycarbonate (PC) being a widely used engineering plastic, its notch and crack sensitivity pose challenges in critical applications. To address this, PC was blended with elastomeric polymers to explore the improvement in toughness. This study systematically investigates the toughening mechanisms of PC blended with acrylonitrile–butadiene–styrene (ABS), copolyether ester elastomer (COPE), and ABS and styrene–ethylene–butylene–styrene (SEBS) copolymer grafted with maleic anhydride (MA). The morphology and mechanical behavior were evaluated under quasi-static and medium-strain-rate tensile tests and Charpy impact tests using optical, electronic, and atomic force microscopy and Raman mapping spectroscopy. The morphological analysis reveals cavitation and crazing phenomena for COPE and SEBS-g-MA systems, and mostly debonding for ABS, indicating an improvement in toughening. While the addition of ABS improves the PC plastic deformation, modifying ABS with maleic anhydride enhances the elastic modulus. Blending PC with SEBS-g-MA increases the strain at break, and the addition of COPE significantly improves the deformation behavior of PC (by around 115%). This comparative study provides valuable insights into the performance of different PC–elastomer blends under similar conditions, supporting the selection of appropriate materials for given applications.

## 1. Introduction

The combination of lightweight, transparency, and heat and high mechanical impact resistance makes polycarbonate (PC) one of the engineering plastics most used in several industries. Despite its excellent properties, PC faces some challenges in critical applications due to its notch and crack sensitivity. This is characterized by the change from plane stress to plane strain conditions at the notch zone, resulting in different failure behavior, from shearing to crazing [1]. Ductile fracture mode is a plane stress condition that mainly occurs in thin bodies, where all stresses are placed on the same plane and the normal stress is negligible. Moreover, in thicker bodies, the brittle fracture mode is associated with a plane strain condition, characterized by zero strain at the normal direction of the crack path caused by triaxial conditions [2]. In critical applications, the tension developed at the impact point (like in PC toe caps in personal protective equipment) could promote a crack initiation on the material. This can, ultimately, result in a catastrophic failure, causing harm to the user even at a low mechanical solicitation [3,4]. In many applications, parts have different shapes and sizes, and the transition from plane stress to plain strain depends on the thickness of the material. In the case of neat PC, this transition occurs between 3.18 and 6.35 mm of thickness [5].

When a polymer has some desired characteristics but lacks in performance, one approach to improve its behavior is through melt blending with a rubbery phase [6]. The incorporation of the rubbery phase can shift the transition of plane stress to plane strain conditions for higher thicknesses, mainly due to the ability to dissipate the absorbed energy though several mechanisms (crazing, cavitation, shear yielding). For critical applications, it is crucial to prevent catastrophic failure. The implications for structural design are significant as it can aid in designing thinner and tougher parts, for example, for toe-cap application [3]. PC blends have been studied in recent decades, mostly to improve its toughness, processability, heat stability, and notch sensitivity. Several authors have reported the incorporation of acrylonitrile–butadiene–styrene terpolymer (ABS) [7,8,9,10,11,12], thermoplastic copolyether ester elastomer (COPE) [5,12,13,14], polydimethylsiloxane [15], and styrene–ethylene–butylene–styrene (SEBS) [12,16,17,18,19] and acrylonitrile–butadiene copolymer [20]. Rubber particles have a toughening effect by cavitation phenomena, which helps to relieve the tension at the notch, allowing the matrix to deform by shearing [21,22]. Furthermore, crack bridging has been reported as a toughening mechanism where crack propagation is delayed or stopped due to the formation of bridges of a secondary polymer phase [23]. With these, authors have claimed to be able to increase the polycarbonate toughness, but it is highly dependent on several factors, such as rubber phase content, compatibility, dispersed particle size, processing conditions, deformation speed, and test temperature. However, while improving impact resistance through melt blending, it is not uncommon to have a negative impact on the tensile strength of the material due to the soft elastomeric phase. Therefore, it is crucial to carefully consider and evaluate the specific requirements and desired outcomes when blending different materials systems.

It is known that several factors, such as temperature and strain rate, are crucial in determining the mechanical performance of materials [24]. The latter is particularly important because the mechanical properties of commercial thermoplastics are typically evaluated under conditions of low strain rate. Extrapolating these properties to dynamic events can lead to incorrect results. Therefore, it is recommended to characterize the material under similar conditions to those encountered in real-world applications. Additionally, analyzing the material at high strain rates allows for exploration of response aspects that may not be visible under quasi-static conditions, providing insight into the physical characteristics of the material’s structure and properties [25,26].

While much of the existing research has been focused on testing specific types of systems, there is limited availability of results that compare different systems under identical conditions. To overcome this issue, the present study builds upon the previous work of Rodrigues et al. [12], which conducted a systematic investigation into the toughening mechanism of PC blended with elastomeric polymers, specifically ABS, ABS-g-MA, COPE, and SEBS-g-MA. The morphological, structural, and thermal characterization of these blends have already been discussed [12], showing how each rubber phase is dispersed within PC matrix, and how this could influence the material properties. It was found that the presence of maleic anhydride (MA) groups on ABS and SEBS improved the interface between phases, most likely due to the reaction between terminal -OH group of PC and MA. As a result, the ABS-g-MA phase detached less than unmodified ABS. Furthermore, contrary to previous reports [5], COPE shows great dispersion and compatibility with PC, with a great impact on PC properties (lower Tg and possible transesterification reaction). Comparing cryogenic fracture of all blends, it was found that the main fracture mechanism for ABS, SEBS-g-MA, and COPE is debonding, cavitation, and fibrillation, respectively. 

The novelty of the present research it to obtain new insight into the evaluation of toughening mechanisms through quasi-static and medium-strain-rate tensile tests and Charpy impact characterization, which are correlated with a more detailed evaluation of the morphology by optical, electronic, and atomic force microscopy, as well as Raman mapping, which complement the previous paper.

## 2. Materials and Methods

### 2.1. Materials

Polycarbonate (Lexan 103R) was purchased from Sabic (Riyadh, Saudi Arabia), ABS (Terluran GP-22) BASF (Ludwigshafen, Germany), maleic anhydride (MA) grafted SEBS (Taipol 7126) was kindly supplied by BYK-Chemie (Wesel, Germany) through TER^®^ AS Chemicals Portugal, and COPE (HYTREL 4069, DuPont) by Biesterfeld Ibérica S.L.U. (Porto, Portugal). MA (99%) was acquired from Acros Organics (Geel, Belgium) and dicumyl peroxide (98%) from Alfa Aesar (Geel, Belgium). ABS-g-MA1 and ABS-g-MA2 were synthesized as described in Rodrigues et al., with a grafting degree of 1.5 and 3.1 wt.%, respectively [12].

### 2.2. Sample Preparation

#### 2.2.1. PC Blending

Different blend compositions of PC with unmodified and modified ABS, SEBS-g-MA, and COPE were prepared (Table 1), following the procedure reported in Rodrigues et al. [12]. The amount of rubber phase was selected through the analysis of different works, choosing the best weight percentage with better toughening. For ABS, 5 and 10 wt.% were chosen, as well as for COPE with 10 wt.% [5,7,8,10]. Grafting with MA with different grafting degrees was selected to evaluate if the number of MA groups would have any impact on the final performance of PC. For SEBS-g-MA, it was found that small amounts of rubber particles had a huge impact on the mechanical properties of PC, and therefore, only 1wt.% was used [16,17].

#### 2.2.2. Injection Molding

For mechanical characterization, miniaturized tensile specimens were injected-molded using a Boy 22A injection molding machine (Dr. BOY GmbH & Co. KG, Neustadt-Fernthal, Germany). The specimens were produced with a flow rate of 5 cm^3^/s and an average barrel and mold temperature of 280 and 80 °C, respectively. Holding pressure was set as 950 bar for 6 s and the cooling time set to 8 s. Impact bars were also injected using an Engel 45T, at 290 °C, 40 cm^3^/s, a holding pressure of 120 bar for 11 s, followed by 15 s of cooling time. After processing, all the specimens were stored and kept in laboratory conditions (21 °C, 50% RH) for at least 48 h prior to mechanical testing.

### 2.3. Material Characterization

#### 2.3.1. Blend Morphology

Scanning electron microscopy was performed using a FEI Quanta 400 (FEI, Amsterdam, The Netherlands) after coating the samples with a thin film of gold-palladium (80–20 wt.%). Raman spectroscopy was performed on a LabRAM HR Evolution system (Horiba France SAS, Palaiseau, France) equipped with a confocal microscope and a 532 nm laser. Each Raman spectrum was recorded with a CCD detector, using a ×100 objective lens, a grating of 800 gr.mm^−1^, a spectral acquisition of 10 s, 3 accumulations for averaging, over a range of 600–1800 cm^−1^ (520.15 cm^−1^ calibration performed with a silicon wafer). Mapping was acquired with a measured laser power of 2.9 mW (to avoid sample degradation) over a 4 × 4 μm^2^ area (0.1 μm step, 1681 points), and LabSpec 6 (v. 6.7.1.10) software for data analysis. Atomic force microscopy (AFM) was performed using a Nano-Observer AFM microscope from CSI Instruments (Les Ulis, France) in resonant mode, coupled with a SPM Probe Model FORT (n-type Si, tip radius < 10 nm, resonance frequency 43–81 kHz, spring constant 0.6–3.7 N/m), scan area 10 × 10 μm^2^ and 1024 × 1024 pixel resolution. The results were analyzed using Gwyddion software (v. 2.65). Raman and AFM samples were prepared using a microtome with a glass knife, cooled with liquid nitrogen.

#### 2.3.2. Quasi-Static and Medium-Strain-Rate Tensile Tests

The stress–strain (σ–ε) curves were accessed using an Instron 5969 (50 kN load cell, Instron, EUA, ISO 527 [27]) and a Zwick-Roell Amsler HTM3712 (20 kN load cell, Zwick-Roell, Ulm-Einsingen, Germany) for quasi-static and medium-strain-rate tests, respectively. The strain at break ($\varepsilon_{r}$) was defined as where the stress decreased to 60% of the maximum stress, the toughness (U) as the integral of the curve, the elastic modulus (E) the initial linear region (crosshead velocity of 1 mm/min for quasi-static test), and the yield stress ($\sigma_{y}$) as the peak stress value of the curve. The crosshead velocity was 50 mm/min (quasi-static) and 1 m/s (medium strain rate), corresponding to a strain rate of 2.5 × 10^−2^ s^−1^ and 30 s^−1^, respectively. The results are an average value of at least 5 dumbbell specimens (33.5 mm grip distance, 20 mm of gauge length, 4 mm and 2 mm of cross-section and thickness, respectively).

#### 2.3.3. Impact Tests

Charpy impact tests were performed to evaluate the impact strength ($a_{CN}$) of unnotched and 2 mm v-notch (type 1A), using an impact test CEAST system with an impact energy of 15 J and rectangular shape specimens (80 × 10 × 4 mm). The tests were performed according to ISO 179-1 [28].

#### 2.3.4. Fracture Morphology

Fracture patterns were evaluated on an optical microscope DMS 1000 (Leica, Wetzlar, Germany) coupled with a polarizer. To visually assess the stress distribution of the specimen after injection molding, a light chamber was utilized with two light polarizer sheets with perpendicular polarization angles.

## 3. Results and Discussion

### 3.1. PC Blends Morphology and Rubber Phase Distribution

**Figure 1:**
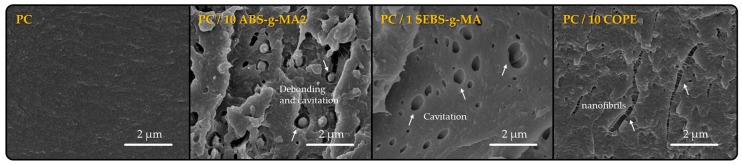
Fragile fracture zone of PC, PC/10%ABS, PC/1% SEBS-g-MA and PC/10% COPE, under electronic microscope at 15,000× magnification (white arrows points to the fracture mechanism).

Figure 1 illustrates the morphology of cryogenic fracture samples, which allows the rubbery phase distribution and interfaces prior to deformation to be evaluated. As mentioned in a previous study, PC exhibits a smooth fracture, indicating a single-phase system. ABS-g-MA2 is distributed throughout the matrix in a spherical shape due to low compatibility. SEBS-g-MA can only be detected by the presence of cavities. It is important to note that a small amount of SBES-g-MA (1 wt.%) can promote cavitation to a greater extent than 10 wt.% of ABS. On the other hand, the COPE phase is indistinguishable from PC [12]. This analysis provides evidence for the main fracture mechanisms observed in each sample: brittle fracture of PC, stress relief through debonding and cavitation in ABS-g-MA2 and SEBS-g-MA particles, and crack prevention through fibrillation in COPE. These findings are valuable for further discussion of the mechanical properties.

**Figure 2:**
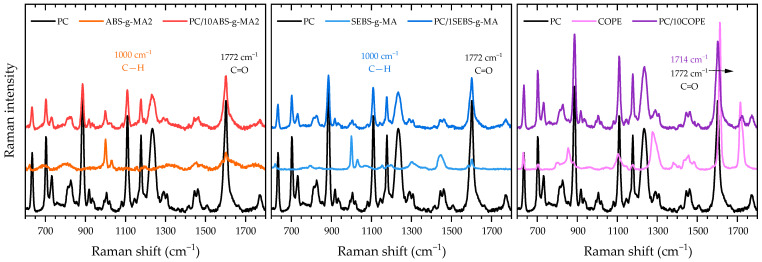
RAMAN spectra of neat PC, ABS-g-MA2, SEBS-g-MA, and COPE, and their respective PC blends. The highlighted shaded rectangles indicate the characteristic bands that were used for Raman mapping.

Prior to conducting Raman mapping, a single spectrum was recorded for each sample (Figure 2) to identify relevant peaks. The most intense peaks of PC were observed at 1601 and 886 cm^−1^, corresponding to the out-of-plane CH wagging mode and ring stretching mode, respectively. Additionally, a broad peak at 1235 cm^−1^ was attributed to C-O stretching, and a peak at 1773 cm^−1^ was attributed to C=O [29]. The spectra of ABS-g-MA2 and SEBS-g-MA were found to be very similar due to the presence of the polystyrene fraction, which could be observed through the CH vibration of the aromatic ring at 1000 cm^−1^ [30,31]. As previously reported by Rodrigues et al. [12], COPE is a copolymer composed of poly(butylene terephthalate) (PBT) and poly(ether glycol) (PEG), with corresponding peaks observed at 1714 (C=O), 1614 (CH), and 1277 cm^−1^ (C-O).

**Figure 3:**
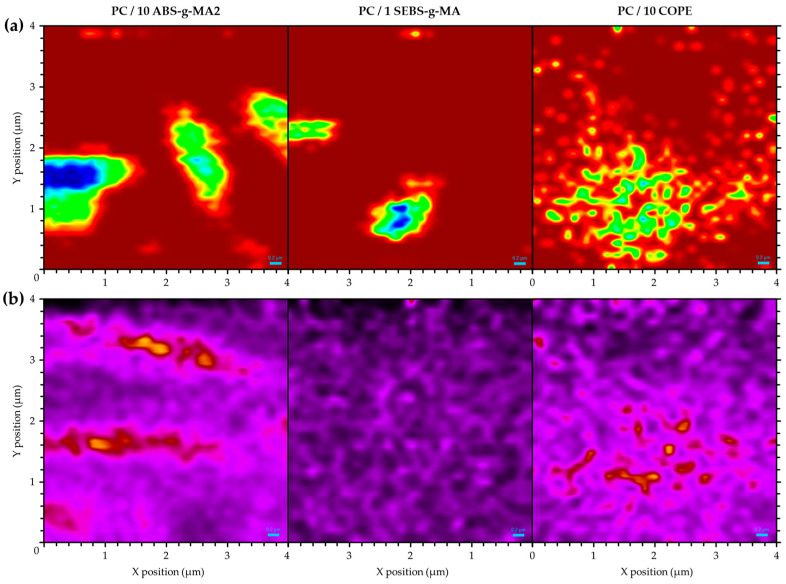
Raman mapping of PC/10 ABS-g-MA2, PC/1 SEBS-g-MA and PC/10 COPE (**a**) at 1772 cm^−1^ and (**b**) area ratio between characteristic bands (dash squares on Figure 1) of dispersed phase (1000 cm^−1^ for ABS-g-MA2 and SEBS-g-MA, and 1714 cm^−1^ for COPE) and PC (1772 cm^−1^). Dispersed phase if represented as (**a**) blue to green and (**b**) yellow to red regions.

Figure 3 illustrates the Raman mapping images of each blend of polymeric systems, where one type of elastomer (ABS-g-MA2, SEBS-g-MA, and COPE) is present in a larger proportion. This visualization provides a clearer understanding of the distribution and interface between the different phases. Two methods were used to characterize the distribution of rubber particles, as described by Huan et al. [32]. In the first method, a distinct PC band (C=O at 1772 cm^−1^) was selected to generate a 2D image that displays the variation in peak intensity, highlighting the regions with a higher concentration of the PC phase. In Figure 3a, the darker regions (blue and green) represent a lower Raman intensity of the PC band, while the red background is attributed to the PC matrix. In the second method, the area ratio between two characteristic peaks (dash squares in Figure 1) was plotted as a function of position (Figure 3b) to obtain a 2D image with better phase contrast (darker regions represent a higher concentration of the PC-abundant phase).

Figure 3a shows circular darker areas with lower Raman intensity for PC/10 ABS-g-MA2 and PC/1 SEBS-g-MA, which could indicate rubbery regions and better dispersion of COPE. However, this method is sensitive to surface topography, and a non-smooth surface could scatter Raman photons differently. SEM images reveal that the systems containing modified ABS and SEBS exhibit a more uneven fracture surface. Additionally, the voids resulting from cavitation or debonding mechanisms can be observed as regions with lower Raman intensity. Using the second method, Figure 3b shows a concentration of rubbery regions with a spherical shape for PC/10 ABS-g-MA2, which is typical for systems with high interfacial tension [33,34]. These particles are the same size as the ones detected in SEM (Figure 1). On the other hand, COPE seems to have a better affinity with the PC matrix with smaller particles and better dispersion.

**Figure 4:**
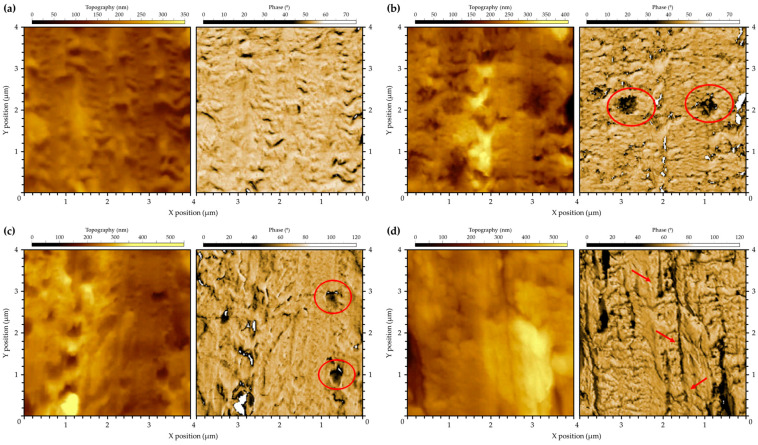
AFM topography and phase images of (**a**) PC, (**b**) PC/10 ABS-g-MA2, (**c**) PC/1 SEBS-g-MA and (**d**) PC/10 COPE blends. Red circles refer to the spherical rubber particles in (**b**,**c**), and red arrows to the dispersed COPE phase in (**d**).

To gain a better understanding of the distribution of the elastomeric phase in the PC matrix, AFM analysis was conducted in resonant mode. This method could assist in identifying materials with varying mechanical properties, such as stiffness, by examining the interaction between the oscillating AFM probe and the sample surface. AFM is a highly effective technique for detecting features at the nanoscale. As SEM was unable to accurately display the distribution of 1 wt.% SEBS-g-MA and 10 wt.% COPE within the PC matrix, AFM could offer valuable insights into the distribution of the rubbery phase in these systems. Figure 4 displays the topography and phase signals for PC, PC/10 ABS-g-MA2, PC/1 SEBS-g-MA, and PC/10 COPE blends. In most samples, the topography surface is irregular, making it challenging to interpret the phase signal [35]. However, when comparing it to the featureless PC phase signal, particles of ABS-g-MA and SEBS-g-MA can be observed dispersed within the PC matrix (Figure 4b,c). Both rubber phases have a droplet shape due to its low compatibility with the matrix, resulting in spherical shapes to minimize interfacial energy. These particles have a similar size to what was observed through SEM (Figure 1) and Raman mapping (Figure 3). The dispersed phase of COPE (Figure 4d) appears elongated along the flow direction and more uniformly distributed, suggesting a lower surface tension compared to the other elastomers. By combining all characterization methods, it becomes evident that ABS-g-MA and SEBS-g-MA have a spherical shape that helps minimize the surface energy, while COPE is more uniformly dispersed with better interaction. This morphology is expected to affect the mechanical and fracture behavior of these blends, which will be further discussed.

Our findings, which are supported by the literature, indicate that no significant structural changes were observed. However, the incorporation of rubbery phases had a major impact on the morphology of PC systems. ABS and SEBS are dispersed as droplet particles due to their incompatibility with the PC matrix. The presence of polar MA groups reduces surface tension, enabling them to form hydrogen bonds with -OH end groups or C=O groups of PC. On the other hand, COPE is well dispersed within the matrix. Although the dispersed phase is not detected by SEM, it can be detected by RAMAN mapping and AFM. The way these phases are dispersed and interact with the PC matrix determines the mechanical behavior of the systems. The droplet shape demonstrates a failure mechanism of cavitation and debonding, while the COPE dispersed morphology exhibits a crazing and fibrillation mechanism.

### 3.2. Quasi-Static and Medium-Strain-Rate Tensile Characterization

**Figure 5:**
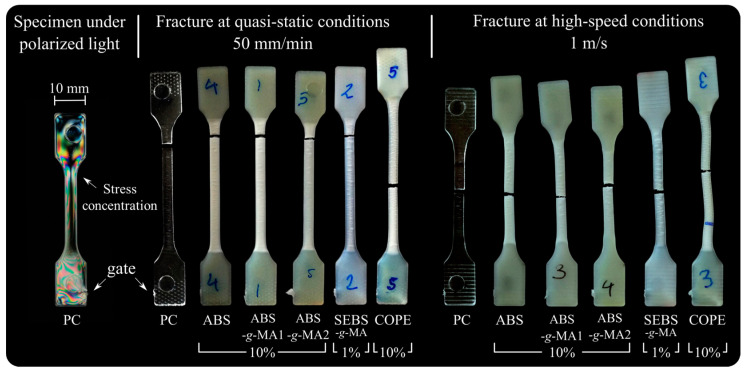
PC tensile dumbbell specimen under two perpendicular polarized sheets on optical microscopy and tested tensile specimen under quasi-static velocity.

**Figure 6:**
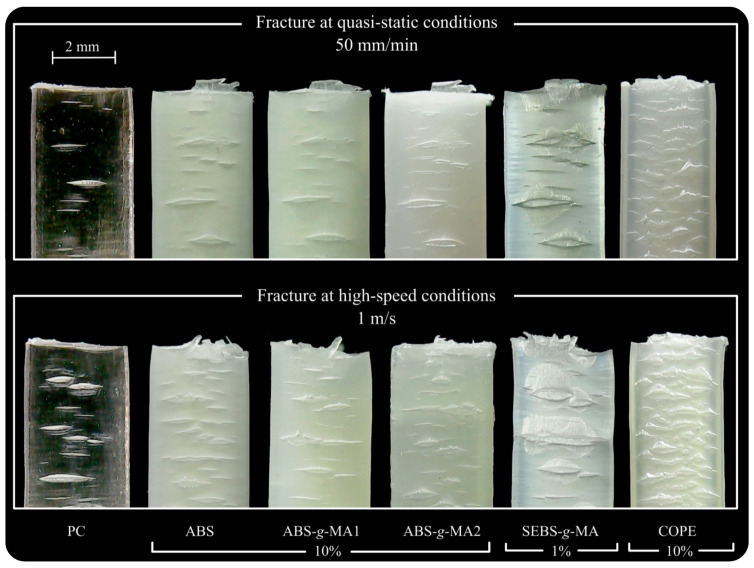
Zone of fracture view from tensile specimens at quasi-static conditions.

Figure 5 and Figure 6 display the morphology of fractured samples following the tensile tests, observed under a light microscope. Table 2 presents the tensile results for neat PC (absolute value) and for PC/elastomer blends (relative value to neat PC). Positive increments in the table are indicated in green, signifying an improvement in the property, while negative increments are shown in red, indicating a deterioration in the property. The strain–stress curves can be found in the Supplementary Materials (Figures S1 and S2). Overall, the quasi-static and medium-strain-rate tensile curves of the tested specimens exhibited a similar trend. Therefore, only the calculated mechanical properties ($\sigma_{y}$, E, $\varepsilon_{r}$, U) are discussed. It is widely recognized that the thermomechanical environment created during injection molding can result in residual stresses. Different flow channel geometries within the part, such as thickness, convergent, and divergent channels, produce a gradient of shear stress. Consequently, the polymer chains align in an anisotropic manner, known as flow-induced stresses. These aligned molecules become “frozen” within the part due to the rapid cooling system of the mold, leading to a non-equilibrium state of the material. Moreover, non-uniform cooling rates can contribute to residual stress phenomena, causing warping in the part (thermal-induced stresses) [36].

The light scattering pattern shown in Figure 5 indicates different molecular arrangements at the top of the gauge length (stress concentration site). Neat PC samples were analyzed by optical microscopy using two crossed polarized sheets in a light chamber to observe how light interacts with a transparent polymer and infer their state of induced residual stress [37]. A comparison with the fractured samples under quasi-static conditions reveals that the preferential fracture zone was indeed observed in this area, except for the PC/COPE blend. During injection molding, all blends (compared to PC) showed a reduction in the injection pressure required to fill the cavity, with the reduction being more pronounced for the blend with COPE. This decrease is related to an increase in the melt’s fluidity, which prevents the formation of critical zones due to residual stress. Additionally, the material’s sensitivity to residual stresses is different at higher strain rates, resulting in a more homogeneous behavior where fracture occurs in the middle section of the specimen. This is because at higher deformation speeds, the material behaves more elastically, as the molecular chains do not have enough time to relax. Therefore, the residual stresses induced during injection molding are less pronounced. In this study, the residual stresses induced by the injection molding process affected the fracture zone of the specimen during quasi-static tests, for all samples except for the PC/COPE system. However, when testing at high-speed deformations, this relationship was no longer observed, and the fracture occurred uniformly at the center of the gauge length. These findings are important for designing parts, as residual stresses can cause product failure, which can be avoided by choosing the right material.

Analyzing Table 2, the addition of ABS does not significantly influence the elastic behavior of PC. However, it does have a positive effect on plastic deformation, as the strain at break increases, especially in medium-strain-rate tests. These findings are in agreement with results reported in the literature [7,8,11]. Additionally, Yin et al. reported a decrease in strain at break with an increasing addition of ABS in quasi-static tests [11]. This variation in behavior can also be attributed to the influence of the ductile to brittle transition of PC, which occurs for specimens with thickness between 3.18 and 6.35 mm and depends on the PC type, molecular weight, and processing conditions [5].

The modification of ABS with maleic anhydride has a positive effect on the elastic modulus of the blends, which becomes more significant with higher amounts of maleic anhydride. Since polybutadiene (PB) is responsible for the flexibility of ABS, the insertion of MA groups onto the PB backbone has a negative effect on this property, making the material more fragile by increasing the elastic modulus (Figure S1). Additionally, the effect of functionalizing ABS is visible even with the addition of small amounts (5 wt.%) of ABS under medium-strain-rate conditions, increasing the toughness up to 6% higher than neat PC. This increase in toughness is due to the dissipation of energy during the deformation and detachment of ABS droplets from the PC matrix, leading to cavitation and crazing of the ABS phase (Figure 1) [38]. Therefore, the influence of ABS modification is more noticeable for lower amounts of ABS incorporation (5 wt.%). The elastic modulus of the system increases, primarily due to the increased rigidity of the ABS phase. This is because MA groups are introduced into the PB backbone, reducing the amount of C=C double bonds. On the toughening side, when comparing all 5 wt.% samples, the modification leads to a greater ability for plastic deformation. This is mainly attributed to the increased compatibility between the ABS and PC phases. This improved compatibility enables better energy transfer under applied loads. It is important to note that ABS is not entirely composed of rubber; the SAN phase is significantly stiffer compared to the PB fraction. Therefore, the degree of cavitation is expected to be less pronounced compared to a droplet composed entirely of rubber, such as SEBS-g-MA.

When 1 wt.% SEBS-g-MA is blended with PC, a small decrease in yield stress and modulus can be observed, while a significant increase occurs in the strain at break, both for quasi-static and medium-strain-rate tests. Although the stress values obtained in the strain-hardening regime for the neat PC curve are always higher until specimen fracture, the PC/SEBS-g-MA blend exhibits a higher tensile strength at fracture (Figure S1). This additional elongation allows the material to absorb more strain energy per unit volume before fracture. Similar results are also found in the literature, where an increasing content of SEBS-g-MA in PC blends is associated with a decrease in tensile strength and tensile modulus, but with a positive effect on the strain at break [17]. Horiuchi et al. show that even with a small amount of SEBS-g-MA, the deformation capability in the tensile tests of PC can be improved by around 18% with a small trade-off in yield stress and elastic modulus [19]. The toughening effect on PC can be explained by the cavitation phenomena observed in SEM (Figure 1) [12]. When a polymer matrix is mechanically stressed, the dispersed rubbery phase within the material can undergo deformation to a certain extent. At this limit, it detaches from the interface, resulting in cavitation and the release of the triaxial stress state. This phenomenon helps to relieve the energy along the material, enabling it to endure greater deformation [17].

The PC/10% COPE blend exhibits mechanical behavior similar to PC/SEBS-g-MA. It shows a decrease in yield stress and a minimal difference in tensile modulus. However, the deformation behavior is clearly different, with a significant improvement of 60% and 114% in the strain at break for quasi-static and medium-strain-rate tests, respectively. Similar to the blend containing SEBS-g-MA, the stress value after yield is always higher in the neat material up to fracture. However, due to the increase in elongation and strain hardening, the stress at break of the blend is higher (Figures S1 and S2). Furthermore, interesting behavior is observed in the PC/COPE blend after the strain value at which the neat specimen fractures. The increase appears to be highly nonlinear with wavy behavior (Figure S2) due to non-uniform deformation and severe warpage (Figure 5). The mechanical properties can be explained by morphological analysis. Both blends show only one phase and exhibit crazing phenomena after fracture, which are associated with improved toughening. The pronounced increase in strain at break leads to a substantial increase in material toughness, possibly due to the presence of fibrils that originate during crazing, as observed in SEM (Figure 1) [12]. After a crack initiates, these fibrils effectively impede its propagation, allowing the material to undergo higher levels of deformation. This behavior is evident in the microscopy images (Figure 6), which show a noticeably higher number of micro-cracks compared to the other blends. As suggested by Rodrigues et al. [12], blending PC with COPE may result in the formation of a copolymer PC-COPE through transesterification reaction. The crack pattern for the PC, PC/ABS, PC/ABS-g-MA, and PC/SEBS-g-MA specimens shows a significantly wider spread and reduced quantity. These results support the observations made in the literature for quasi-static tensile tests [5].

### 3.3. Impact Tests and Fracture Behavior 

**Figure 7:**
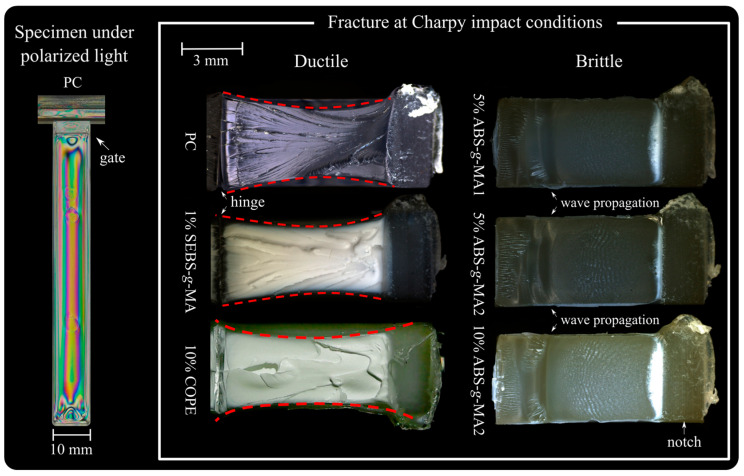
Specimen fracture morphology under impact for ductile (neat PC, 1%SEBS-g-MA, 10%COPE) and brittle (ABS-g-MA) samples.

Figure 7 depicts the bar specimen used for impact tests under polarized light. This reveals a uniform distribution of residual stress throughout the geometry. Based on the findings from tensile tests, the samples selected for impact strength analysis were PC/SEBS-g-MA and PC/COPE, which have the potential to increase the toughness of PC. Additionally, PC/5%ABS-g-MA1, PC/5%ABS-g-MA2, and PC/10%ABS-g-MA2 were utilized to assess the impact behavior in relation to different grafting degrees.

The impact test results presented in Table 3 indicate that none of the elastomers exhibited higher values of the impact strength than neat PC. This observation can be attributed to the discrepancy in the number of processing cycles between the neat PC and the blends. Notably, the neat PC was only injected for testing purposes, whereas the blends underwent multiple processing cycles. Consequently, it is plausible that the impact value of the neat PC, given an equivalent number of processing cycles, could be lower, while the elastomers may potentially show a positive effect.

When comparing the same amount, 10 wt.%, COPE has a lesser impact on the impact strength compared to ABS-g-MA2 for v-notch samples. This result aligns with SEM and tensile analysis, which revealed a promising toughening mechanism through fibrils. However, when assessing the results from the unnotched samples, no significant variation was observed compared to the neat PC. These findings indicate that the performance of these materials is affected by the presence of notches, as it exerts a considerable influence on their performance.

The fracture analysis of the impact shows that neat PC exhibits a fully ductile failure behavior, as evidenced by the reduction in specimen thickness due to plastic deformation (dash line in Figure 7). On the other hand, the addition of ABS-g-MA mainly imparts a brittle behavior to PC (no thickness reduction phenomenon was observed), while SEBS-g-MA and COPE maintain a fully ductile fracture. 

**Figure 8:**
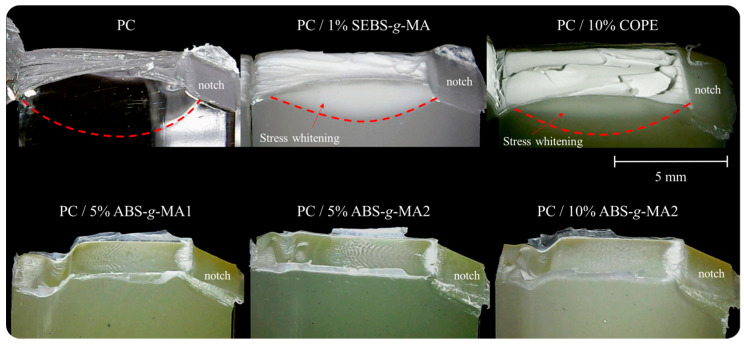
Specimen fracture side view under impact for ductile (neat PC, 1%SEBS-g-MA, 10%COPE) and brittle (ABS-g-MA1 and ABS-g-MA2) samples.

For the systems containing ABS-g-MA, the crack initiates with a local stress whitening area, while for the others, it occurs throughout the deformed area in the perpendicular plane of the crack propagation. By observing the crack fronts of PC/ABS-g-MA from a different angle (Figure 8), it is possible to detect crack propagation consistent with brittle behavior, changing direction when it is about to reach the opposite side of the specimen (lower propagation velocity). This behavior was also reported by Aranda-Ruiz et al., where the brittle fracture of PC is associated with fracture mode I [39]. 

Before the wave propagation zone, the surface is smooth and clear, transitioning into a more irregular morphology at the end of the crack propagation. Additionally, when the fracture was complete, no hinge was observed, while for neat PC, PC/SEBS-g-MA, and PC/COPE, the fracture was not complete. These results may be related to the thickness of the samples (around 4 mm), as the transition from PC’s plane stress (ductile) to plane strain (brittle) lies between 3.18 and 6.35 mm thickness [5]. 

It should also be noted that PC, PC/1% SEBS-g-MA, and PC/10% COPE exhibit significant deformation following impact. This deformation results in thickness reduction and stress whitening, which can be attributed to the excellent deformation capability of the matrix material, as already discussed in Section 3.1.

## 4. Conclusions

The results of this scientific article focused on the toughening mechanisms of polycarbonate (PC) blended with elastomeric polymers, namely ABS, ABS-g-MA, COPE, and SEBS-g-MA provided valuable insights into the behavior of PC blends and shed light on the influence of different elastomeric phases on the material’s performance.

The analysis of the mechanical properties revealed that adding ABS to PC had a positive effect on plastic deformation, as shown by an increase in strain at break. Modifying ABS with maleic anhydride further improved the elastic modulus of the blends. Inserting MA groups into the ABS backbone increased the material’s fragility but also enhanced its toughness. Incorporating SEBS-g-MA in PC blends resulted in a decrease in yield stress and modulus but significantly increased the strain at break. This improvement in deformation capability can be attributed to the cavitation phenomena observed in scanning electron microscopy images. Additionally, AFM and Raman mapping showed that COPE is better dispersed than modified ABS and SEBS, with lower surface tension. This helps explain the higher toughness of these blends under quasi-static and medium-strain-rate deformations. The PC/COPE blend exhibited similar behavior to the PC/SEBS-g-MA blend, with a decrease in yield stress and minimal difference in tensile modulus. However, the strain at break showed a substantial improvement, indicating enhanced deformation capability. The presence of crazing phenomena and fibrils in SEM images suggested an improvement in toughening mechanisms. Furthermore, the fracture behavior of PC/COPE was independent of the residual stresses induced during injection molding.

The selection of PC blend systems should be based on the desired mechanical properties and specific applications. If a stiffer PC sample is required, PC/ABS systems are preferred to increase the elastic modulus. However, for applications that require higher toughness and the ability to withstand greater plastic deformation, PC/SEBS and PC/COPE systems are more suitable choices. In general, PC/ABS systems are recommended for structural applications where static loads are predominant due to their enhanced stiffness. On the other hand, PC/SEBS and PC/COPE composites are better suited for energy absorption applications, such as those involving dynamic impacts, free-falling objects, bumpers, or shields, due to their superior toughness and ability to endure significant plastic deformation.

Overall, the study highlights the potential of melt blending elastomeric polymers with PC to enhance its mechanical properties, particularly toughness. The findings contribute to the understanding of the toughening mechanisms in PC blends and provide valuable insights for the development of advanced engineering plastics for various applications. Furthermore, these findings help improve our understanding of the practical implications of using each material system in specific applications. Future research can further explore the optimization of blend compositions and processing conditions to achieve the desired material properties for specific applications.

## Figures and Tables

**Table 1:** Polycarbonate blends’ compositions.

Composition [wt.%]	PC	ABS	ABS-g-MA1	ABS-g-MA2	SEBS-g-MA	COPE
PC	100	-	-	-	-	-
PC/5 ABS	95	5	-	-	-	-
PC/10 ABS	90	10	-	-	-	
PC/5 ABS-g-MA1	95	-	5	-	-	-
PC/10 ABS-g-MA1	90	-	10	-	-	-
PC/5 ABS-g-MA2	95	-	-	5	-	-
PC/10 ABS-g-MA2	90	-	-	10	-	-
PC/1 SEBS-g-MA	99	-	-	-	1	-
PC/10 COPE	90	-	-	-	-	10

**Table 2:** Variation * in PC properties at quasi-static and medium-strain-rate tests.

	PC	ABS		ABS-g-MA1		ABS-g-MA2		SEBS-g-MA	COPE
	100	5	10	5	10	5	10	1	10
Quasi-static									
$\sigma_{y}$ [MPa]	64	0%	1%	1%	2%	2%	4%	2%	2%
E [GPa]	1.8	4%	2%	0%	7%	4%	10%	1%	1%
$\varepsilon_{r}$ [%]	55	8%	10%	2%	9%	2%	10%	18%	60%
U [MPa]	30	7%	9%	4%	9%	1%	12%	19%	59%
Medium strain rate									
$\sigma_{y}$ [MPa]	78	3%	3%	3%	4%	3%	5%	2%	2%
E [GPa]	3.1	4%	9%	2%	3%	7%	11%	1%	5%
$\varepsilon_{r}$ [%]	41	11%	33%	3%	34%	1%	31%	47%	114%
U [MPa]	27	9%	37%	6%	40%	1%	37%	47%	111%

* Property variation: no variation, positive variation, or negative variation. Standard deviation is lower than 5%.

**Table 3:** Impact strength of notched and unnotched specimens.

Specimen	Impact Strength (kJ/m^2^)	
	v-Notch	No Notch
PC	94 ± 4	272 ± 26
PC/5% ABS-g-MA1	21 ± 2	265 ± 8
PC/5% ABS-g-MA2	16 ± 2	268 ± 7
PC/10% ABS-g-MA2	17 ± 1	254 ± 6
PC/1% SEBS-g-MA	78 ± 2	259 ± 2
PC/10% COPE	89 ± 1	277 ± 3

## Data Availability

The data presented in this study are available in the article.

## Supplementary Materials

The following supporting information can be downloaded at: https://www.mdpi.com/article/10.3390/polym16162303/s1, Figure S1: Representative engineering stress–strain curves of quasi-static tests of PC, PC blends, ABS and ABS-g-MA2.; Figure S2: Representative engineering stress–strain curves of medium-strain-rate tensile tests of PC, PC blends and ABS.
