# Supplementary material for: Investigation of Toughening Mechanisms in Elastomeric Polycarbonate Blends through Morphological and Mechanical Characterization at Small and Medium Strain Rates
Source: Polymers (Basel). 2024 Aug 15;16(16):2303. doi: 10.3390/polym16162303 (PMC11359002; doi:10.3390/polym16162303)
Supplement: Supplementary file 1 [file polymers-16-02303-s001.zip › polymers-3062576-supplementary.pdf]

polymers

MDPI

Supplementary Material

Investigation of Toughening Mechanisms in Elastomeric Polycarbonate Blends through Morphological and Mechanical Characteriza-tion at Small and Medium Strain Rates

Pedro Veiga Rodrigues *, Bruno Ramoa, Maria Cidália R. Castro and Ana Vera Machado

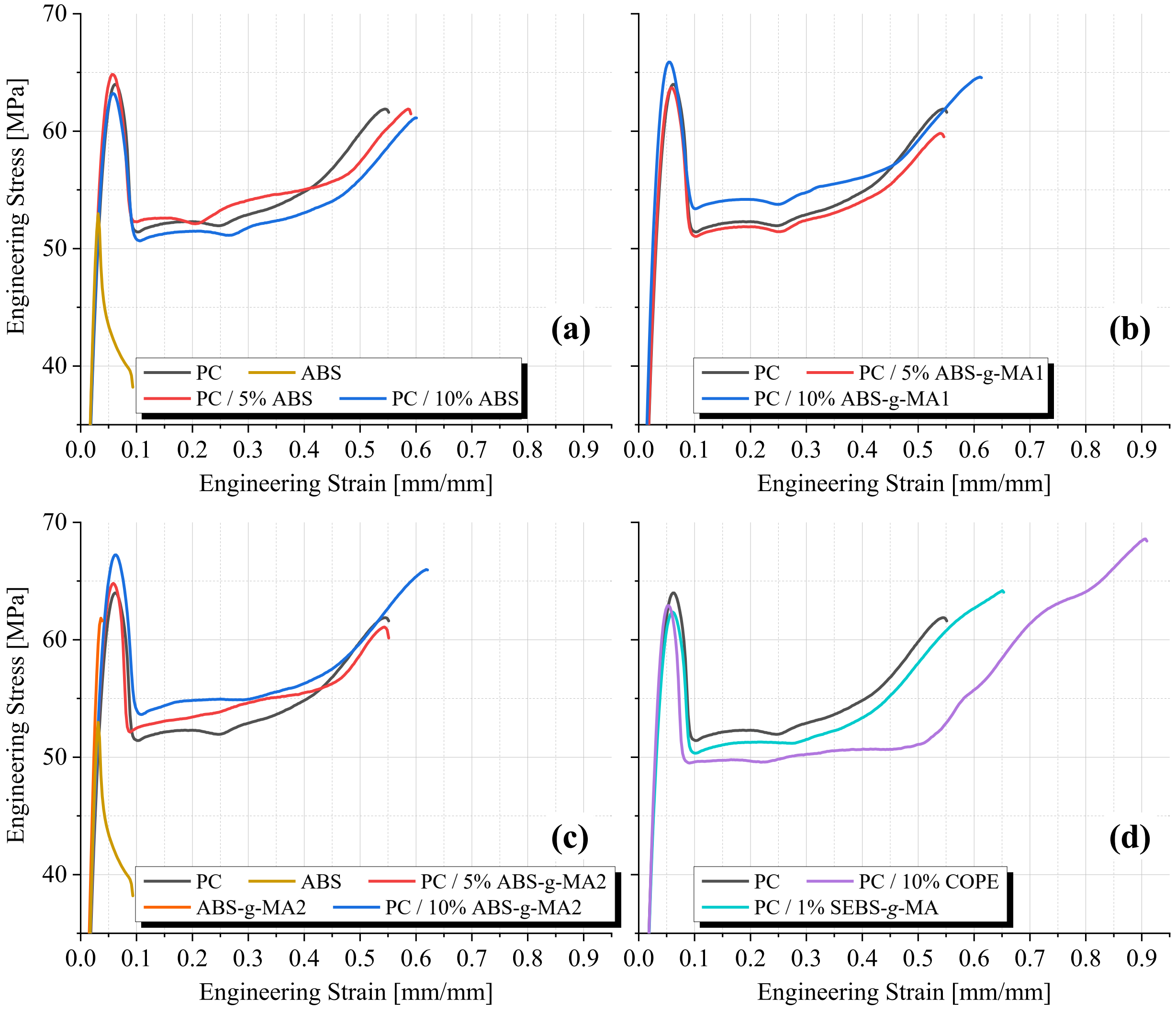

Figure S1: Representative engineering stress-strain curves of quasi-static tests of neat materials: PC, ABS and ABS-g-MA2; and PC blends: "PC / 5 ABS, PC / 10 ABS, PC / 5 ABS-g-MA1, PC / 10 ABS-g-MA1, PC / 5 ABS-g-MA2, PC / 10 ABS-g-MA2, PC / 1 SEBS-g-MA, and PC / 10 COPE.

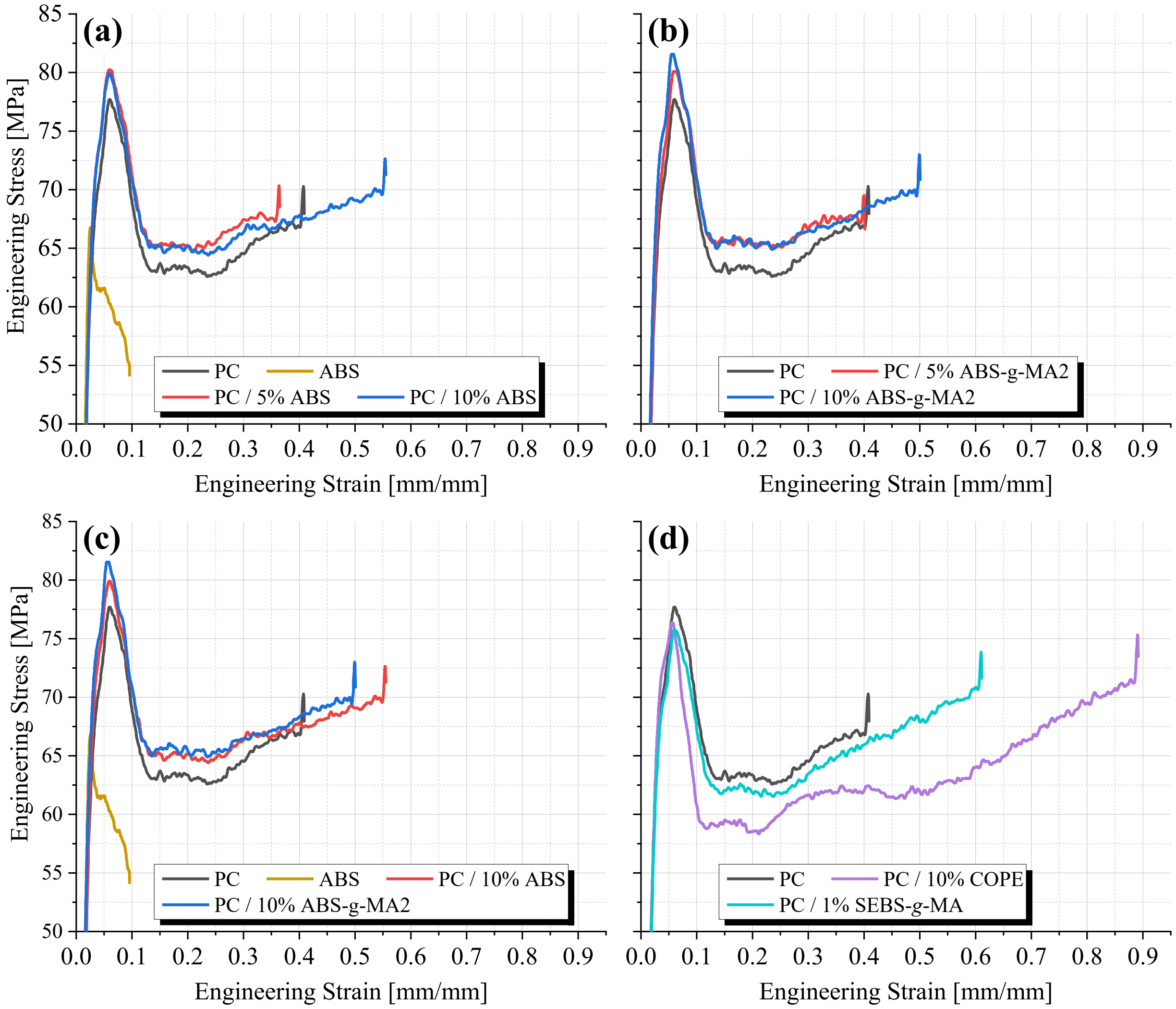

Figure S2: Representative engineering stress-strain curves of medium strain rate tensile tests neat materials: PC and ABS; and PC blends: "PC / 5 ABS, PC / 10 ABS, PC / 5 ABS-g-MA2, PC / 10 ABS-g-MA2, PC / 1 SEBS-g-MA, and PC / 10 COPE.
